# Genomics research in Africa and its impact on global health: insights from African researchers

**DOI:** 10.1017/gheg.2018.3

**Published:** 2018-06-08

**Authors:** N. S. Munung, B. M. Mayosi, J. de Vries

**Affiliations:** 1 Department of Medicine, University of Cape Town and Groote Schuur Hospital, Cape Town, South Africa; 2 Dean's Office, Faculty of Health Sciences, University of Cape Town, Cape Town, South Africa

**Keywords:** Africa, genomics medicine, genomics research, global health equity

## Abstract

Africa may be heading for an era of genomics medicine. There are also expectations that genomics may play a role in reducing global health inequities. However, the near lack of genomics studies on African populations has led to concerns that genomics may widen, rather than close, the global health inequity gap. To prevent a possible genomics divide, the genomics ‘revolution’ has been extended to Africa. This is motivated, in part, by Africa's rich genetic diversity and high disease burden. What remains unclear, however, are the prospects of using genomics technology for healthcare in Africa. In this qualitative study, we explored the views of 17 genomics researchers in Africa on the prospects and challenges of genomics medicine in Africa. Interviewees were researchers in Africa who were involved in genomics research projects in Africa. Analysis of in-depth interviews suggest that genomics medicine may have an impact on disease surveillance, diagnosis, treatment and prevention. However, Africa's capacity for genomics medicine, current research priorities in genomics and the translation of research findings will be key defining factors impacting on the ability of genomics medicine to improve healthcare in Africa.

## Introduction

Genomics medicine will likely be a key component of future healthcare. It is tipped to make significant contributions to the diagnosis, treatment and prevention of diseases [1]. There are already examples of how genomics has made its way into mainstream medical practice and public health in low- and middle-income countries (LMICs) [2]. A case of interest is a recent change in the HIV management policy in Botswana where the country opted out of efavirenz-based therapies as first-line anti-retroviral therapy (ART), in favour of dolutegravir [3]. Genomics studies had showed that about 13.5% of the Botswana population are unable to effectively metabolize efavirenz-based therapies [4].

Overall, there is heightened enthusiasm on the role genomics could play in reducing global health inequities, especially following the completion of the human genome project [5] and the complete sequencing of African genomes [6]. Despite this enthusiasm, some have cautioned that much still needs to be done before genomics medicine will be a reality in Africa and eventually contribute to improving global heath equity [7–10]. The promise of most genomics and biobanking initiatives in Africa is to overcome some of these challenges; to extend the genomics revolution to Africa; and to use genomics as a tool to reducing global health inequities [11–13].

Currently, the use of genomics in the clinical setting in Africa is dominated by the genotyping of single genes with the aim of elucidating genetic aetiologies for single-gene disorders such as sickle cell anaemia and Downs syndrome [2, 14]. Even so, this service is still largely unavailable in many healthcare settings in Africa. There is almost unanimous agreement that understanding population genetic variation as it links to health is the gateway to genomics medicine in Africa. As a result, a number of population genomics studies are ongoing in Africa. As part of a broader study on the benefits and risks of genomics research in Africa, we asked researchers working in genomics in Africa what they consider to be some of the health benefits of genomics to African patients; the availability of genomics medicine in their countries and the challenges for genomics medicine in Africa.

## Methods

We performed a qualitative research study to explore the potential role of genomics research in the future of healthcare in Africa. The aim was to interrogate the future of genomics medicine in Africa and the role genomics research could play in reducing global health inequities.

We conducted in-depth interviews with 17 African professionals who were actively involved in genomics research that focusses on African populations. Our study adopted a grounded theory design [15]. Purposive sampling [16] was used to select research participants. We sent email invitations to a number of researchers who were involved in ongoing genomics projects in Africa, inviting them to participate in the study. The research professionals interviewed in this study were based in institutions in eight different countries representing the different geopolitical regions in sub-Saharan Africa (i.e. Central, East, West and Southern Africa). The details of the study population has been previously described [17].

In the interviews, we asked participants if they had an example of how genomics was being used in the healthcare setting in their home countries or other African countries; what they envisage to be some of the ways genomics could quickly be integrated into clinical practice in Africa; and the challenges and opportunities for genomics medicine in Africa.

All interviews were audio recorded and transcribed verbatim by NSM. Transcripts were imported into NVivo10 to facilitate data analysis. The first round of coding was done by NSM using two interview transcripts. The codes and themes emerging from the first round of coding was discussed with JdV. This led to the development of a coding framework that was then applied to all the transcripts. The results of the thematic analysis can be discussed in three broad categories: current uses of genomics in the clinic; possible uses of genomics in Africa and the challenges of genomic medicine in Africa.

## Results

This study was based on the premise that genomics research has the potential to reduce global health inequities and that Africa is heading for an era of genomics medicine. For some interviewees, genomics medicine is already making vital contributions in improving healthcare in Africa, even though it is still unavailable in many African countries. Although a majority of the interviewees could not immediately think of examples of the clinical application of genomics in healthcare in their countries, they were of the opinion that in the not so distant future, there would be clinical applications of genomics in Africa, especially with regards to improved diagnosis of disease conditions.

There are many examples, in the United States. I am from Country X. I don't know if there is a direct application of genomics in Country X. What I do know is that sickle cell disease is a genetic disease that has various components to it and some of the genetic findings about diagnosis, screening, management of the disease are being applied to variable extents in different parts of Country X depending on the resources, at least in the area of diagnosis. (R-01)

However, a few of the researchers interviewed mentioned that genomics tools are only sparingly available in the clinical setting in Africa and the most cited example was the diagnosis of sickle cell anaemia, a monogenic condition that is common in much of sub-Saharan Africa and responsible for high morbidity and mortality rates in infants and children [18]. The few interviewees who mentioned examples of the application of genomics in healthcare in Africa cited diagnosis and prenatal screening for sickle cell disease and Downs syndrome.

We had started genetic diagnosis before for Disease A and for chromosomal abnormality in Country X in 2009 and we are able to say if a child is affected with sickle cell anaemia from three months of pregnancy, affected with Down syndrome from three months of pregnancy.(R-05)

In the example quote above, the interviewee was referring to the use of genomics technology in an urban public health facility. Very few countries in Africa currently offer comprehensive genetic services and where it exists, it is mainly in urban areas and in tertiary healthcare institutions [19] and therefore unavailable to a majority of the population in these countries. Given the scarcity of examples of the application of genomics in healthcare in Africa, we asked the researchers what areas of genomics may easily make an impact on healthcare in Africa in the short term. Three broad areas emerged: improved diagnosis, pharmacogenomics and public health.

### Disease diagnosis

One of the most direct ways in which interviewees perceived that genomics can impact on healthcare in Africa is in improved diagnosis for diseases and in the prediction of risk of complex diseases. Generally, advances in genomics have facilitated testing for monogenic conditions [20]. Genomics is also fast becoming a crucial diagnostic tool for infectious diseases [21]. In the interviews, most African genomic researchers indicated that genomics would improve disease diagnosis in Africa, especially for multifactorial genetic conditions. The most frequent examples given were in the diagnosis of cystic fibrosis and breast cancer.

One of the direct benefit has been in designing new diagnostics that are appropriate for mutations that occur in African populations. One example is cystic fibrosis which everybody thought only occurred in European populations but we have shown that it also occurs in African populations and the mutation profile is different. So it's very important that in Africans you test the correct mutation profile, so that will be one benefit from the diagnostic point of view (R-03)

One interviewee also mentioned that genomics could facilitate diagnosis of rare diseases for which there are no available diagnostic tests. In fact, in 2008, the US National Institute of Health (NIH) established the undiagnosed diseases programme with the aim of advancing knowledge on the use of genomics such as exome sequencing, to improve molecular diagnosis of rare and common diseases using genomic technologies [22].

## Pharmacogenomics

The second aspect in which the researchers expected genomics to make an impact on health care in Africa is in pharmacogenomics. Pharmacogenomics is the study of how an individual's genetic make-up may influence their response to a particular drug. It combines knowledge of pharmacology (the science of drugs) and genomics (the study of genes and their functions) with the goal of improving the efficacy, safety and dosing of medications. The proportion of patients who respond to treatment using current drugs ranges from 25% to 80% [23] and it is predicted that genomics could remedy this situation through improved drug targeting [20] – a view that was echoed by all the researchers that we interviewed.

If you think of pharmacogenetics, studies of a certain population might show, for example, that certain variation affecting the metabolism of a drug is in high prevalence in this population and it could indicate that may be the drug shouldn't be used or should be used in a lower dose to be effective, so at the population level, this might be applicable directly. (R-04)

Frequently cited examples of how the application of pharmacogenomics could rapidly impact on healthcare in Africa was in the management of HIV and hypertension at the population level (and not necessarily at the individual level).

By looking at differences in disease progression, particularly with HIV, we are looking at what is the genetic basis for children who are infected at birth and they don't get symptoms until age 10 or older versus rapid progressors, which are children who are born with HIV and who develop the disease within 6 months or so. And so by teasing out those genetic reasons, we may be able to improve their treatment or prophylaxis to delay onset of HIV or to target HIV prevention at very genetic levels and stages of disease progression. So not necessarily personalized medicine but definitely, the methodologies that will be geared towards sub-Saharan African children. (R-02)

There is already evidence of the application of the results of this kind of genomics studies to healthcare in Africa. In 2016, for example, Botswana changed its first-line HIV treatment policies from efavirenz-based therapies to dolutegravir after studies showed that approximately 13.5% of the population in Botswana had two copies of the gene variant responsible for slow metabolism of efavirenz [3, 24]. Infact, pharmacogenomics studies have shown that the genetic allele CYP2B6*6, which causes slower rates of metabolism for several drugs, including efavirenz, is higher in African populations than in Caucasians [25, 26].

## Public health genomics

A third way African researchers expected genomics to impact on healthcare in Africa relates to public health – specifically, disease surveillance and prevention. Although not an obvious link – genomics medicine is most frequently cast in the light of personal medicine – in the African context, the expectation is that the benefits of genomics research could most easily be extended to the population level. The use of genomics in public health interventions is transforming modern infectious disease surveillance and the investigation of disease outbreaks [27]. Generally, genomics has transformed the way in which public health professionals fight epidemics and pandemics for high profile infectious diseases such as tuberculosis, HIV, influenza, salmonellosis and Ebola [27–29]. In our study, the researchers who mentioned this short-term application of genomics use in healthcare in Africa frequently referred to how genomics was instrumental in the fight against the 2014 Ebola outbreak in West Africa.

When there is an outbreak of a pathogenic bacteria in the clinical setting and, there is a sequence to track where it is coming from and how it has spread, that knowledge helps in control just in the same way they have used sequencing to track how the Ebola virus has mutated and spread. It is clinical, it is not just for research purposes. (R-11)

## Challenges for genomics medicine in Africa

### Cost of genomics tools

Whilst many of our interviewees were optimistic that there are realistic ways in which genomics could impact on healthcare in Africa, they also expressed some doubts on the potential application of genomics medicine in the near future in Africa. Many were of the opinion that populations in Africa have burning healthcare needs that may not necessarily require expensive approaches or technologies. They however argued that this was not to suggest that genomics medicine is not important, but rather that the cost-benefit analysis of genomics technologies is opposed to standard methods of healthcare.

There are so many pressing needs for healthcare in Africa which are much less expensive and are more a priority in terms of pneumonia, TB, HIV, malnutrition, infant mortality, those are the kinds of things which are urgent healthcare needs. The cost effectiveness of genomic interventions will be much higher than the cost of many basic interventions like childhood vaccination. So it's probably not going to result in massive changes in the short term but I think it is going to start educating practice at tertiary healthcare settings. But it's a long term goal. (R-12)

The high cost of genomics medicine coupled with the near absence of regulatory frameworks for genomics medicine in Africa was also considered a serious challenge to the implementation of genomics medicine in Africa. As one of our interviewees said, Africa would need to carefully consider the economics of genomics and the ethical issues raised by its introduction into healthcare in Africa.

You have to talk about the ethics or the willingness to spend an equivalent of 50 000 dollars on one patient, when you think that, that could probably buy vaccines for 40 000 people. (R-11)

Similar views have also been echoed by others who also argue that LMICs will have to consider the economics of genomics, its impact on healthcare in LMICs and its contribution to global health inequities before they embark on the journey towards making genomics medicine available for all [30].

The affordability of genomics medicine has been a centre of debate for many years [31–33] and it is argued that the high cost of genomics technology may limit access to genomic medicine in LMICs [34]. Whilst some of our interviewees were of the opinion that cost may be a deterrent for the use of genomics medicine in Africa. All interviewees who mentioned cost as a challenge to genomic medicine in Africa also said this should not discourage genomics research and biobanking efforts in Africa.

For me, many people will not afford it because we do not have universal health coverage like many western countries. So it is a very expensive commodity. But the point is that should not deter us from trying to understand, the role of genetics in disease causation which ultimately would help, even at least for people who can afford. (R-10)

There was considerable disagreement on the perceived high cost of genomics medicine and the impact it may have on access to genomics medicine in Africa. Whilst there appears to be an agreement that genomic medicine is relatively expensive, the cost of genomic technology is going down [1, 20]. Some interviewees described that just like other innovations that were once thought to be expensive, as technology becomes more and more available, genomics technology will become affordable.

I think at the moment, African patients are not likely to be able to pay for that. But in time it will become cheaper I don't think we must be put off by those issues now, people had said that for ARVs 20 years ago, when antiretroviral were in the hundreds of thousands of Rands or dollars a year. People said there is no way it could be used in Africa. Now, we could still be in that position and I think we need to press ahead and trust that efficacy and technology will get us there. (R-12)

Whilst it is important to be optimistic, it is important to keep in mind that if genomics medicine becomes unaffordable or not accessible to the majority of the population in LMICs, then the purpose of using it as a tool to reduce global health inequities would have been defeated.

### Translation of genomics research

If health research is to be used as a tool for reducing global health inequities, the translation of research findings into clinical practice is essential [35, 36]. The same has been argued for genomics research in Africa [37]. Although it may seem too premature to kick start discussions on the translation of genomics research in Africa to clinical benefits, it may actually be time to move from promise to practice (Urban, 2015).

Fears of the high cost of translating the outcomes of genomics research has led to concerns that genomics research in Africa may result in exploitation of LMIC populations who take part in research and yet may not be able to access genomics medicine. In our study, most interviewees expressed the need for a translational phase in African genomics research and were of the opinion that it would require public–private partnerships.

The scientific community who knows the value of this, the clinicians who need this to treat their patients better, can constitute an advocacy or pressure group and approach international bodies who are already aware of the advantages of this and convince their governments to support these initiatives. (R-06)

However, translation of research results to useful clinical products or services involves innovation, patenting and securing intellectual property rights for the innovation. This has the tendency to increase cost and limit access.

When it comes to translational research, those who have greater capacity to do that sort of research will benefit most because the results of basic research is shared, but the products of more translational research tend to be protected; intellectual property rights, patents and if there is a product, there is the risk that those participating in the research might find themselves less able to afford these things because their countries are less able to afford the more specific translational research. (R-04)

To overcome the high cost of translating genomics research, some authors have suggested that researchers communicate and engage with key stakeholders [38, 39]. This may involve engaging healthcare providers, policy makers and the general public on discussions on the provision of genomics services for certain healthcare uses and the positive impact it may have at the population level compared to other available or recommended methods of diagnosis, treatment and/ or prevention.

### Focusing genomics research in Africa on Africa's health priorities

If the goal of genomics research in Africa is to advance the ideals of global health justice through reducing global health inequities, then, like other forms of global health research, genomics research projects in Africa should ideally target public health problems in Africa [7]. This view was shared by a majority of the interviewees. They argued that if the results of genomics research in Africa are to be translated to useful healthcare applications, genomics research in Africa would have to focus on Africa's health priorities rather than targeting a broad range of diseases

It [genomic research] has to be directed at the problems of public health interest to Africa. We need to align with our healthcare priorities and identify issues where there may be problems. I guess what we should probably be worried about is doing research which probably doesn't meet our needs or just touching on tiny fractions of the disease. (R-12)

Some researchers have argued that genomics research in Africa is more likely to make an impact if it focuses more on infectious diseases [27, 40–42]. We asked our interviewees what could be possible areas of focus for genomics research that increase the potential impact of genomics on healthcare in Africa. Whilst all the researchers indicated that genomics should focus on diseases prevalent in Africa, commonly cited examples were HIV, Malaria and tuberculosis.

I really do think that we should have something that is mostly disease oriented and population genomics oriented. I think there is lot of population genomics going on now, but I think it should be disease oriented and probably disease that are specific, severe and frequent on the African continent example is HIV, another example is TB. I think anything that really is a huge burden. I am not saying that the others are not important. They are important but it is just the scale of priorities. (R-05)

In other LMICs in Asia and Latin America, genomics research has had great impact in the diagnosis of microbial and parasitic infections such as leishmaniasis and dengue fever [43].

### Capacity building for genomic medicine in Africa

A third challenge for genomics medicine that emerged from the interviews is the limited capacity for genomic medicine in healthcare settings in Africa. The future of genomics medicine in Africa would, to a large extent, be determined by the availability of the required capacity [1, 7, 9, 25]. All interviewees identified the lack of adequate healthcare infrastructure for genomics technology as a major barrier to introducing genomics medicine in the clinic in Africa. This includes limited infrastructural capacity for genomics testing in most African healthcare settings.

It is going to be the capacity to do the testing, right, so you have to put the laboratory infrastructure. The reality is that diagnostic laboratory infrastructure in most Africa countries is very poor, there are exceptions to that but that is the case in many poor African countries and even in some of the rich African countries like Nigeria, it is not fantastic. So laboratory infrastructure needs to be in place. (R-12)

It also included limitations in human resources for genomics medicine.

Part of the issue is not whether you have genomics in the clinic. Part of it is whether it can be used in the clinic. If the manpower exists to use the technology. Not every country has the manpower. So, you will also need to have trained staff and that is going to take quite a while, It's not like the experts are so many, you need to train a whole lot more. Otherwise, you end up with a situation where people actually do have fantastic machines but with no one to use them. You heard the presentation, when someone was talking about having a powerful HPLC machine and how it is not being used because there is no one to use it. (R-11)

The future of genomics medicine in Africa would, undoubtedly, depend on the availability of highly skilled clinical geneticists in Africa [1, 7, 9]. A survey on the understanding of concepts in genetics by healthcare professionals who were based in an African country revealed that most general practitioners had limited understanding of concepts in genetics [8]. Africa would therefore benefit from capacity-building efforts directed at training medical geneticists, genetic counsellors and laboratory technicians/technologist in the fields of molecular diagnosing, and some have suggested that building capacity for genomics medicine should go hand in gloves with capacity building for genomic research in Africa [9].

### Public education in genomics medicine

For Africa to harness the health benefits of genomics medicine, it is important that genomics research consortia in Africa incorporate activities geared at building capacity for genomics medicine. One way to achieve this is to develop and implement educational programmes for healthcare practitioners and the public at large [7, 8]. In the interviews, some African researchers were of the opinion that public education in genomics medicine could indirectly lead to government buy-in and investment in genomics medicine.

We first have to educate them about what is pharmacogenomics. You cannot engage a person if they don't understand what it is. How do you convince them to invest? Most of the politicians in Africa, most of the time, they do not understand this. You have to engage them through education. (R-16)

Currently the focus on education in genomics is primarily directed at health researchers with a number of key stakeholders such as policy makers, healthcare practitioners and the general public being left out of such capacity building initiatives.

## Discussion

Global health research consortia in Africa can advance the ideals of health justice through: setting research priorities that are of benefit to worse-off populations; building capacity for health research; and having a translation plan on how the outcomes of genomics research may be used to influence policy and practice [36, 44]. In our study, African researchers were also of the opinion that if genomics medicine is to become part of healthcare in Africa or to be used as a tool to reduce global health inequities, then genomics research and biobanking consortia in Africa will need to: fine tune their research agenda to reflect the health needs of populations in Africa; articulate a translation phase or plan for the translation of research findings; and build capacity for genomics medicine in Africa.

In our study, African genomics researchers mentioned the need for genomics research in Africa to be tailored to the health priorities of the host countries. This may be a challenge for genomic research consortia in Africa, especially as most African countries are yet to articulate their health and health research priorities. It is therefore hard to pitch genomics research on priorities that have not been identified. The researchers also mentioned that genomics would have more impact on health in Africa if it adopts a disease-oriented approach at the population level and if it focuses on diseases that are major contributors to the disease burden in Africa. Whilst the WHO has statistics on diseases burden in Africa and communicable and non-communicable diseases dominate this list [45], there is little information on which health conditions should be prioritised in the case of genomics research in Africa. H3Africa, for example, has adopted a disease-focused approach to population genomics research in Africa, and current projects are addressing the genetics of communicable and non-communicable diseases [12]. However, what is unclear is if the research agenda for genomics projects such as those within H3Africa are in line with national health and health research priorities of African countries. Nevertheless genomics is already having an impact in public health in Africa as demonstrated during the 2014 Ebola outbreak in West Africa [29] and in the management of HIV-positive patients in Botswana [3].

Genomic medicine is no doubt gaining applicability in the clinical setting in LMICs. Yet, as the results of this qualitative study suggest, there is very limited capacity for genomics medicine in Africa. Previous research work has documented that very few physicians in Africa understand concepts in genetics [8] and that the number of trained genetic counsellors in Africa is alarmingly low [14]. What may seem pressing at the moment is the need to build capacity for genomics medicine in Africa. Effective capacity building in genomics medicine will need to be more strategic and may benefit from a needs assessment to identify what is available and what needs to be done. This is essential in avoiding duplication and making maximum use of limited resources.

Capacity building in genomics medicine in Africa should cut across infrastructural and skills building. A few institutions and initiatives in Africa offer training in genomics medicine for different healthcare professionals. Some universities in Africa offer post graduate training in genetic counselling and a small number of programmes exists in Africa for the training of medical geneticists. However, these training programmes are still very limited and more will need to be done to establish formal training programmes in genomics medicine in Africa. This should span training of physicians, laboratory technologists, genetic counsellors and nurses. Our interviewees suggested that this could be done alongside genomics research in Africa. H3ABioNet, a project within the H3Africa initiative, recently launched an online course in genomics medicine for African-based healthcare professionals (https://training.h3abionet.org/AGMC_2016/course-description/). This course, which was first rolled out to nurses using a blended learning approach, will also be extended to other healthcare professionals in Africa. However, more genomics medicine initiatives are needed in Africa if genomics medicine is to become part of clinical care in Africa.

Arguably, the promise of genomics to be a valuable resource for the future of medicine and healthcare in Africa lies more in the translation of research results to practical health tools. Genomics initiatives in Africa are encouraged to have a plan for translation research results to policy and practical health tools. Feedback of genetic test results is one aspect that should receive attention at the moment. Genomics research projects in Africa should consider exploring ways of reporting incidental findings and genetic test results to patients in ways that are socially and culturally acceptable to the populations in question. Empirical studies on feedback of genetic test results or incidental findings could help direct policy development for genetic testing and reporting in Africa.

The world needs to harness the power of genomics to reduce global health equities. Advances in genomics and genomics medicine is relatively slow in LMICs and may wonder if people in LMICs will access the benefits of genomics. In our study, African researchers and clinicians. There is a need for genomics research consortia in Africa to conduct a need assessment for genomics medicine in Africa and to identify ways in which the outcomes of genomics research in Africa could be practically integrated into health care for the benefit of African populations.
